# Time-Induced Progressive Alteration of Kir Current in Cerebral Smooth Muscle Cells of Stroke-Prone Spontaneously Hypertensive Rats

**DOI:** 10.1155/2013/849750

**Published:** 2013-04-23

**Authors:** Michèle Bastide, Thavarak Ouk, Olivier Pétrault, Régis Bordet

**Affiliations:** ^1^Department of Pharmacology, EA1046, University of Lille Nord de France, 59045 Lille Cedex, France; ^2^IUT A, University of Lille Nord de France, 59653 Villeneuve d'Ascq Cedex, France; ^3^LBHE, EA2465, University of Artois, 62307 Lens, France

## Abstract

We investigated the involvement of potassium inward rectifier current (Kir) impairment in smooth muscle cells of cerebral arteries under the condition of increased susceptibility of stroke, in spontaneously hypertensive stroke-prone (SHRsp) rats compared to spontaneously hypertensive (SHR) ones as well as to controls (WKY). Kir current was studied with whole-cell patch-clamp techniques on freshly isolated single smooth muscle cells (SMC) of middle cerebral artery (MCA) from SHRsp, SHR, and WKY male rats (are range 12–32 weeks). A significant and progressive Kir current density reduction was observed on SMC of SHRsp rats from the 22nd week of age on, as opposed to the Kir current density stability observed over the same time in the SMC of WKY and SHR rats. The Kir density alteration was correlated to the age of the SHRsp animals. These results suggest that in the cerebral vascular smooth muscle cells of SHRsp rats, there is a progressive Kir channel impairment, leading to a reduction of Kir current density. This impairment may underpin a lack of vasodilation of the MCA and be implicated in the stroke-proneness observed on SHRsp animals.

## 1. Introduction

K^+^ channels of arterial smooth muscle cells (SMC) play a major role in the control of the vascular tone by their regulation of the cell membrane potential. The opening of K^+^ channels allows a repolarization of SMC membrane, a closure of voltage-dependent calcium channels that leads to a relaxation of arteries. In the SMC membrane of middle cerebral arteries (MCA), four types of potassium channels have been described [1]: (i) voltage-gated K^+^ channels, K_v_; (ii) calcium-activated K^+^ channels, K_Ca_; (iii) ATP-sensitive K^+^ channels, K_ATP_; (iv) barium-sensitive inward rectifier K^+^ channels, Kir.

The Kir channel is specific of small diameter arteries and constitutes a K^+^-induced vasodilation system with the Na^+^/K^+^-ATPase pump [2]. Very low extracellular concentrations (1 to 5 mM) of K^+^ produce a transient vasorelaxation by activation of Na^+^/K^+^-ATPase specifically blocked by ouabaïn. Following neuronal activation, K^+^ ions are released by neurons allowing an extracellular K^+^ concentration ranging from 7 to 15 mM. K^+^ ions siphoned by astrocytes that are released close to cerebral vessels by the astrocyte processes, and a sustained vasodilatation developed in response to the increase of Kir current density [3, 4]. Hence the K^+^ ions released during neuronal activity constituted a link between the cerebral metabolism and the cerebral blood flow.

In a pathological condition like chronic hypertension, ionic transport systems may be impaired and contribute to the deleterious relationship between hypertension and stroke. Prestroke electromechanical alterations as well as a loss of cerebral autoregulation have been identified in SHRsp demonstrating that hypertension is involved in both occurrence and severity of stroke [5, 6]. This relationship has been modeled in SHR rats, more susceptible to cerebral ischemia, and in SHRsp, a strain that spontaneously develops cerebral ischemia. McCarron and Halpern [7] demonstrated an alteration of the Ba-sensitive Kir-dependent vasodilator mechanism in cerebral arteries of SHRsp rats, whereas the Na^+^/K^+^-ATPase mechanism was intact or even slightly increased. The occurrence of a Kir channel-dependent relaxation impairment is of particular interest, as these channels are indeed drastically impaired in cerebral ischemia. An impaired vasodilation of the MCA due to a reduced activity of Kir channels could increase the severity of cerebral lesions by preventing an optimal reperfusion of the tissue [8]. We further studied this phenomenon in particular on MCA myocytes after ischemia/reperfusion and its correlation to the volume of the cerebral infarct [9]. 

We hypothesized that a prestroke alteration of Kir channels activity could be involved in the occurrence and severity of cerebral ischemia in SHRsp. We undertook measurements of Kir current densities by whole-cell patch-clamp technique on myocytes dissociated from MCA of SHRsp, WKY, and SHR rats over 20 weeks. 

## 2. Materials and Methods

All experiments were performed in strict accordance with guidelines of the National Institutes of Health, the French Department of Agriculture, and the local ethics committee of Nord-Pas de Calais. Ten-week-old male SHR and Wistar-Kyoto rats were purchased from Elevage Janvier (France) and Iffa Credo (France), respectively. Four-week-old SHRsp rats were kindly offered by Max-Delbrück-Centrum für Molekulare Medizin (D-13125 Berlin, Germany). All rats were housed in a temperature-controlled environment (20 ± 1°C) and maintained on a 14 h light/10 h dark cycle with *ad libitum* access to food and water.

Blood pressure was measured by tail cuff using a LE5001 rat-tail blood pressure system (LSI Letica, Panlab, Spain).

 All experiments were done on freshly dissociated cerebral vascular SMC, extracted from MCA of WKY, SHR, and SHRsp animals. MCA myocytes were obtained by an enzymatic procedure previously reported [9], and Kir current was assessed by the whole-cell patch-clamp technique [10]. The pipettes (2.7 to 3.3 MΩ) were filled with the pipette solution that contained (in mM) 130 KCl, 2 MgCl_2_, 10 EGTA, 10 HEPES, 5 phosphocreatine, and pH 7.3. The bath solution contained (in mM) 134 NaCl, 6 KCl, 1 MgCl_2_, 0.1 CaCl_2_, 10 HEPES, and pH 7.4. All assays were run at room temperature (19–22°C). The standard voltage clamp protocol consisted of 185 ms-voltage ramps from −140 to +50 mV, from a HP of −60 mV. The difference between the transmembrane current and the current recorded in presence of 0.5 mM Ba^2+^ (a specific inhibitor of Kir channels) unmasked the Kir current [9, 11]. The current density (pA/pF) was calculated and preferentially used to compare the different cells to account for cell size variability.

All values were expressed as mean ± standard error of the mean (s.e.m.). Kir current densities were compared with one-way ANOVA followed when ANOVA is significant by a post hoc protected least significance difference (PLSD) Fischer test. A value of *P* < 0.05 was considered significant.

## 3. Results

### 3.1. Systolic Blood Pressure and Body Weight of the Rats

**Figure 1:**
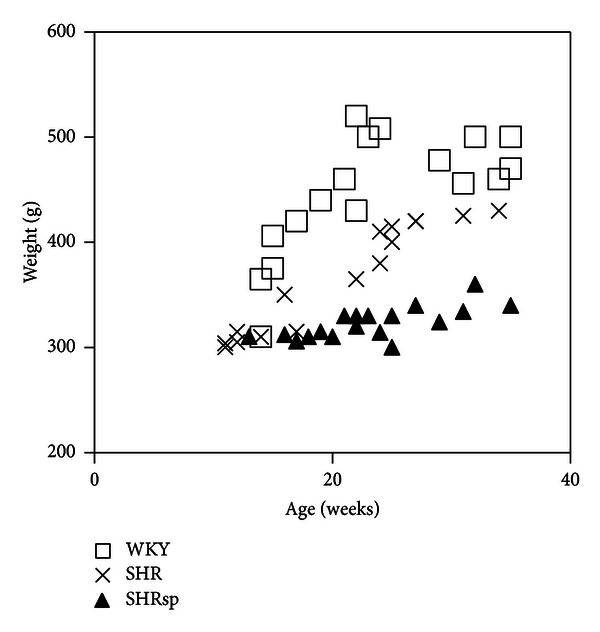
Body weight evolution from 11 to 35 weeks old for WKY (*n* = 17), SHR (*n* = 16), and SHRsp (*n* = 18) rats.

 The animals were studied from week 12 to week 32. Systolic blood pressure was measured over the 3 first weeks. Higher systolic blood pressure (SBP) was significantly established (*P* < 0.05) for SHR and SHRsp in comparison with WKY rats. The SBP values were 142 ± 4 mm Hg (*n* = 7), 232 ± 9 mm Hg (*n* = 7), and 206 ± 4 mm Hg (*n* = 6) in WKY, SHR, and SHRsp, respectively. The profile of body weight evolution was rather different between WKY and SHRsp: before 22 weeks of age, 396.57 ± 19.21 g (*n* = 7) for WKY and 313.29 ± 2.97 g (*n* = 7) for SHRsp; from the 23rd week on, 482.20 ± 8.89 (*n* = 10) for WKY and 329.27 ± 4.67 g (*n* = 11) for SHRsp (Figure 1). SHR weights were positioned in an intermediate range.

### 3.2. Kir Current Densities in Vascular Smooth Muscle Cells of WKY, SHR, and SHRsp

**Figure 2:**
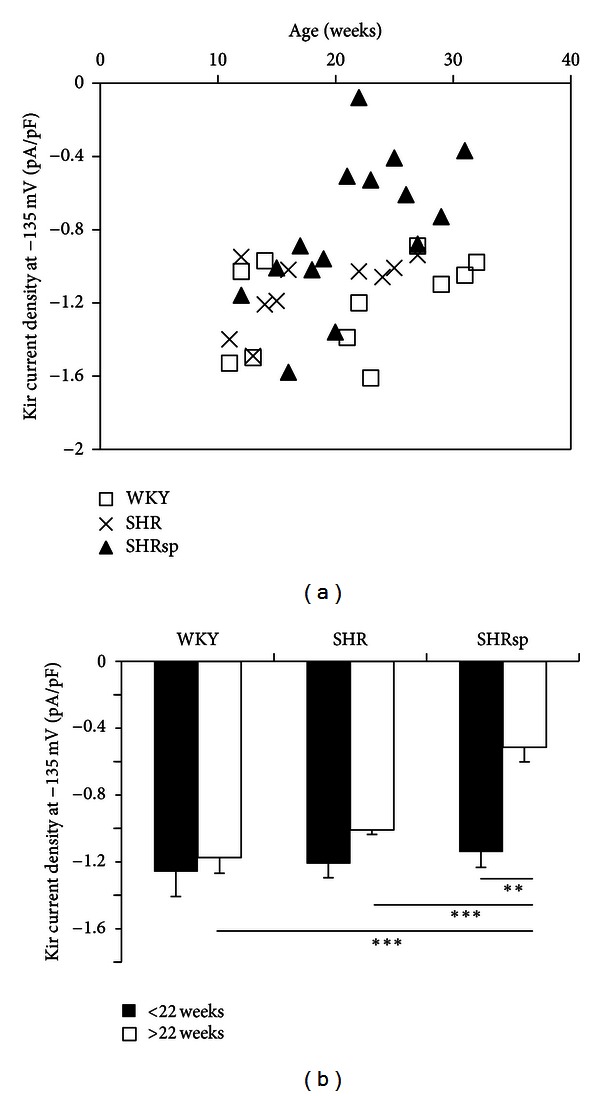
(a) Kir density measured at –135 mV on SMC originated from WKY (*n* = 11), SHR (*n* = 10), and SHRsp (*n* = 15) rats at different ages. (b) Histogram of Kir densities measured at –135 mV for WKY, SHR, and SHRsp for the period 12–22 weeks and the period 22–32 weeks. Values are mean ± sem, ***P < 0.000, **P < 0.005.

 Current responses to voltage ramps were recorded in myocytes extracted from the MCA of WKY, SHR, and SHRsp animals at different ages. Current densities acquired for each myocyte at −135 mV, potential at which Kir amplitude was maximal, were extracted from the ramp responses, averaged for the myocytes issued from the same animal and plotted according to the age of the animal (in weeks) for WKY, SHR, and SHRsp. The capacitance values did not differ among groups: 20.88 ± 0.39 pF (*n* = 11 rats, *n* = 26 cells) for WKY cells, 18.06 ± 0.27 pF (*n* = 10 rats, *n* = 21 cells) for SHR cells, and 19.22 ± 0.31 pF (*n* = 15 rats, *n* = 30 cells) for SHRsp cells. At the beginning of the study, Kir densities for the three groups were collected. Overtime, Kir current densities for WKY and SHR rats did not show significant variations as opposed to the ones obtained on SHRsp vascular myocytes where a reduction was observed from the 22nd week, as illustrated in Figure 2(a).

 Up to week 21, SHRsp Kir densities ranged from −1.58 to −0.89 pA/pF and from −0.73 to −0.008 pA/pF afterward (Figure 2(b)). There is a significant difference (*P* < 0.005) between SHRsp before and after week 22, as well as between SHRsp after week 22 versus WKY and SHR whatever the time period (*P* < 0.000). Interestingly, there was no difference among the three groups prior to week 22.

### 3.3. Time Evolution of Kir Current Densities in Vascular Smooth Muscle Cells of SHRsp

**Figure 3:**
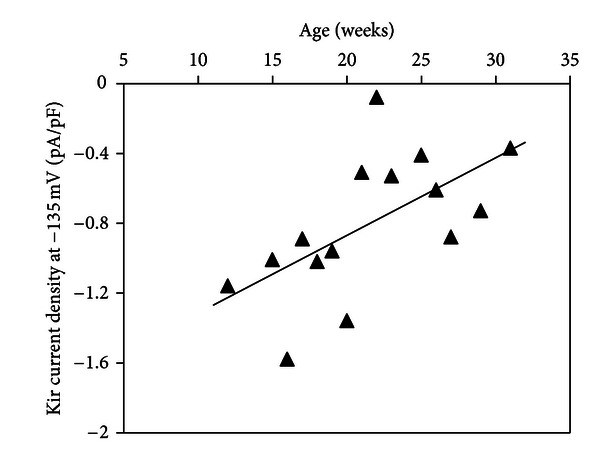
Age-related alteration of Kir current density (pA/pF) measured at −135 mV in vascular smooth muscle cells of MCA of SHRsp rats (*n* = 15). Solid line represents first-order linear regression (correlation coefficient *r* = 0.587,  *P* < 0.05).

The Kir current densities measured at −135 mV on SHrsp myocytes, plotted versus age in Figure 3, are negatively correlated (*r* = 0.587,  *P* < 0.05). 

## 4. Discussion

By use of patch-clamp recordings on vascular myocytes, our study shows that the density of the Kir current involved in the K^+^-dependent vasodilation of rat's small cerebral arteries is altered in the SHRsp animals through aging, when compared to age-matched SHR and WKY rats. The reduction of the Kir density is time dependent, appearing progressively along the study in the SHRsp strain. Age-matched WKY and SHR rats showed no modification of Kir densities on cerebral vascular myocytes over the same period although high blood pressure was present in SHR rats.

 This Kir density reduction has been suggested by McCarron and Halpern [7] who demonstrated by vasomotion experiments on MCA segments that the K^+^-dependent vasodilation following a slight increase of K^+^ concentration (superior to 7 mM) was attenuated or even absent in MCA of SHRsp. On male SHRsp aged 16–25 weeks, they found an impaired K^+^-dependent dilation in 7 of 8 arteries tested. In physiological conditions, the membrane potential of vascular smooth muscle cells allowed the K^+^ ions to flow out of the cell by Na/K-ATPase and Kir channels activation to initiate repolarization of smooth muscle cells and vasodilation of the arteries. Potassium ions released following neuronal activation are siphoned through glial cells to maintain normal nonhyperexcitable extracellular K^+^ concentration and released by astrocytes processes surrounding the arterial wall resulting in a local dumping of K^+^. Local K^+^ increase activated Kir current inducing the Kir vasodilation and a necessary increase of local blood flow to answer to neuronal energy [4, 12]. The reduction of Kir current density resulted in a reduced Kir-dependent vasodilation and in an impairment of metabolic autoregulation of brain in SHRsp compared to SHR or WKY rats. A lack of energy supply for the neuronal tissue could be very detrimental and prevented the optimal activity of neurons. This Kir alteration specifically observed in aged SHRsp but not in the age-matched SHR could participate to the proneness and the occurrence of spontaneous ischemia observed in SHRsp since the progression of Kir impairment is concomitant to the appearance of spontaneous stroke in SHRsp rats. Different reports demonstrated that SHRsp developed stroke within 22–24 weeks of age and before; no cerebral lesion was detected while the severe hypertension was already developed. The regional cerebral blood flow was progressively reduced in specific brain regions where stroke preferentially occurred [5, 13, 14]. Compared to WKY, SHRsp displayed a greater ischemic damage when stroke is induced by occlusion of MCA. In normotensive rats, Kir current density has been shown to be significantly reduced after stroke resulting in an extent of ischemic lesions as the relationship between infarct volume and reduction of Kir density has been evidenced [8]. Hence it is likely that in SHRsp the Kir channel impairment must be potentiated since a reduction was already present in the prestroke phase, as described here. It can be hypothesized that an additional poststroke alteration would more or less suppress the neurovascular coupling. After ischemia, the ensuing disruption of the delivery of substrates to brain cells would contribute to brain lesion extent.

Chronic hypertension is known to properly induce a remodeling of vessels (reduction in vessel cross-sectional area, increase of wall thickness, and wall/lumen ratio) due to both blood pressure-dependent and blood pressure-independent components. A loss of pressure-dependent constriction and a dysfunction of NO synthases have been described in MCA of SHRsp both leading to an impaired perfusion in SHRsp rat [15]. Among blood pressure-independent factors, oxidative stress, particularly the O_2_°^−^ anion, seemed to play an essential role. In hypertension O_2_°^−^ overproduction saturates the physiological antioxidant systems and its accumulation induced endothelial as well as muscular dysfunctions. Oxidative stress was very deleterious for ionic channel activities as free radicals could interact with amino acids residues of channel proteins [16]. In heart, potassium conducting protein channels were profoundly affected in structure and function by the redox state particularly by a modification of sulfhydryl radicals of cysteine residues [17, 18]. The defect of Kir activity here observed could derive from an altered function of channels due to oxidative stress. It has been demonstrated that the development of stroke proneness in SHRsp is preceded by numerous dysfunctions of cerebral vessel including endothelium alteration, inflammatory response, plaque instability, and majored oxidative stress [19, 20]. Antioxidant treatment during the prestroke phase could be protective. Pires et al. [21] showed that daily treatment of SHRsp with an antioxidant, tempol, prevents vessels remodeling, independently from blood pressure. Recently cardiovascular protective effects of statins or fibrates treatment in SHRsp have been demonstrated towards endothelial dysfunction, inflammation, and oxidative stress on cardiovascular functions [22, 23]. In a normotensive rat model of focal ischemia/reperfusion, a significant reduction of Kir density in ischemic SMC was evidenced due to a major oxidative stress occurring at the reperfusion step since the administration of antioxidant agents, completely prevented the decrease of Kir density and concomitantly reduced infarct volumes [9, 11, 24]. 

## 5. Conclusion

We here showed that in SHRsp MCA smooth muscle, an age-dependent reduction of Kir current density occurs, as opposed to age-matched SHR and WKY. Kir channels alteration and the consequent Kir-dependent vasodilation impairment could participate to a progressive, time-dependent dysfunction of neurovascular coupling. This prestroke vascular dysfunction would contribute to the stroke-proneness, pointing to vascular protection as a strategic therapeutic target in the prevention of stroke in patients with high risk factors.
